# Prevalence of *Theileria annulata* in dairy cattle in Nyala, South Darfur State, Sudan

**DOI:** 10.14202/vetworld.2017.1475-1480

**Published:** 2017-12-15

**Authors:** Ismail A. Abaker, Diaeldin A. Salih, Lima M. El Haj, Rawia E. Ahmed, Manal M. Osman, Awadia M. Ali

**Affiliations:** 1 Department of Parasitology, Faculty of Veterinary Science, University of Nyala, Nyala, Sudan; 2 Central Veterinary Research Laboratory, P.O. Box 8067, Al Amarat, Khartoum, Sudan; 3 Department of Parasitology, Faculty of Veterinary Medicine, University of Khartoum, P.O. Box, 13314 Khartoum-North, Sudan

**Keywords:** indirect fluorescent antibody test, polymerase chain reaction, South Darfur, Sudan, *Theileria annulata*, ticks

## Abstract

**Aim:**

This study was conducted in dairy cattle in Nyala, South Darfur State, during the period from June to September 2015, to study the prevalence of bovine tropical theileriosis.

**Materials and Methods:**

Apparently, healthy cattle of different age groups, different breeds, and from both sexes were randomly selected from seven locations. Three age groups of cattle were selected, group one <1 year old, group two 1-3 years old, and group three older than 3 years. These cattle were indigenous and cross (Friesian X zebu). A total of 150 blood samples were collected for blood smears, blood in EDTA tubes, and serum samples as well as ticks infesting cattle. Three diagnostic techniques were used such as blood smear, indirect fluorescent antibody test (IFAT), and polymerase chain reaction (PCR).

**Results:**

Of 150 samples, 11 (7.3%, 95% confidence interval [CI]: 9.1-5.5) were positive for *Theileria* spp. piroplasms in the blood smears, 70 (46.7%, 95% CI: 35.7-57.7) were positive for *Theileria* *annulata* antibodies in the IFAT, and of 100 samples, 39 (39%, 95% CI: 46.6-31.4) were positive for *T. annulata* using PCR. The prevalence of *T. annulata* was higher in indigenous breed than cross cattle by the three diagnostic techniques. The highest prevalence of *T. annulata* was recorded among cattle older than 3 years old. There were three genera and ten species of ticks found feeding on cattle. These were *Rhipicephalus evertsi evertsi, Rhipicephalus decoloratus, Rhipicephalus annulatus, Hyalomma dromedrii, Hyalomma impeltatum, Hyalomma rufipes, Hyalomma anatolicum, Hyalomma truncatum, Amblyomma variegatum*, and *Amblyomma lepidum*.

**Conclusion:**

The study concluded that tropical theileriosis is prevalent among dairy cattle in Nyala. *H. anatolicum* was found in very low numbers, suggesting other ticks may play a role in the transmission of the disease. Molecular characterization of *T. annulata* is recommended for accurate mapping of the disease and evaluates the magnitude problem of tropical theileriosis in South Darfur region.

## Introduction

Tick-borne diseases (TBDs) are widespread in Sudan causing substantial economic losses and are a constant threat to the development of animal wealth [1]. In the Sudan, tick fauna comprises over 70 species prevalent in diverse ecological zones [2].

Tropical theileriosis is a parasitic disease caused by the hemoprotozoan *Theileria annulata* that is transmitted to cattle by ixodid ticks of genus *Hyalomma* [3]. Two stages in the life cycle of the parasite are responsible for the pathogenesis of the disease. These are schizont in mononuclear cells of the reticuloendothelial system and intraerythrocytic piroplasm [4]. The disease occurs in a wide zone of Africa, Southern Europe, and a large part of Asia [5]. It is the most important TBDs in Northern Sudan, with 14% of cattle in this region are infected with *T. annulata*, but later serological tests using indirect fluorescent antibody assay (IFA) and ELISA suggest that the prevalence is much greater (over 30%) [6]. Latif [7] reported that 85% of farms investigated in Khartoum experienced clinical theileriosis and mortality of 22% and 30% in young calves and heifers, respectively.

In Sudan, livestock population constitutes 40 million cattle, 50 million sheep, 42.5 million goats, 4 million camels, and 0.5 million horses [8]. South Darfur State in Western Sudan is one of the richest states concerning animal population that is estimated to be 11 million consisting of 3.9 million cattle, 3.6 million sheep, 2.9 million goats, 53500 donkeys, 30700 horses, and 8700 camels [9]. Livestock farming is an important component of the agricultural sector in the Sudan for the provision of animal-based food products and as a source of income for resource poor farmers. Jaafer [10] reported that tropical theileriosis is endemic and its vector is prevalent in Nyala, with 31% of cattle had antibodies against *T. annulata* infection in the region using IFA test (IFAT).

Limited studies were conducted to determine parasitological, serological, and molecular prevalence of tropical theileriosis in South Darfur State. The objective of this study was to determine parasitological, serological, and molecular level prevalence of tropical theileriosis among dairy cattle in Nyala, South Darfur State, Sudan. Moreover, an update distribution status of tick’s species infesting cattle in Nyala, South Darfur State, was provided.

## Materials and Methods

### Ethical approval

The study reported here was carried out in strict accordance with the recommendations in the standard operating procedures of the University of Nyala. We confirm that the samples were collected after receiving specific approval from animal owners.

### Study area

**Figure-1:**
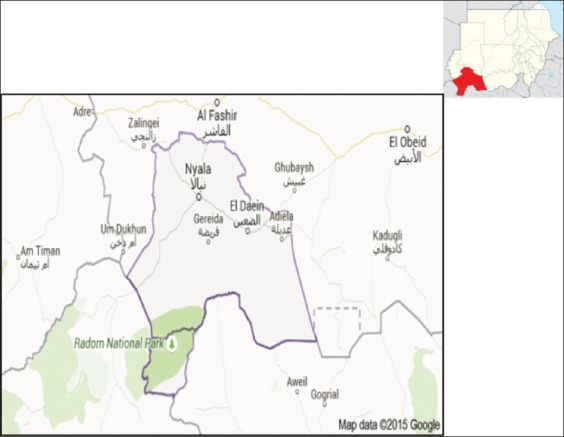
Map Sudan (top) showing South Darfur State and map of South Darfur State showing Nyala city.

This study was carried out in Nyala, South Darfur State. It is located in western parts of the Sudan and lies between latitudes 8°30′ to 13°′N and longitudes 23°15′to 28°E (Figure-1). Seven locations along the city were selected, these were Museah in the East-South, Al shabi and Karary (South), Al gabal (East), Alseraif (South-west), Almawashi (North), and Algeer (West).

### Collection of samples

In each location, samples were collected from apparently healthy cattle from eight farms that were set apart. The samples (n=150) collected were blood smears, serum, and whole blood in EDTA as well as ticks. The breeds of cattle were indigenous (Butana and Kenana) and cross (Zebu X Friesian). Age groups were <1 year old, between 1 and 3 years old, and more than 3 years old.

### Blood smears examination

Blood smears were stained with 10% Giemsa^’^s stain and examined under 100× oil immersion objective using light microscope for the presence of *Theileria* spp. piroplasms. At least 50 microscopic fields were examined, and the presence of one or more piroplasm was considered positive.

### IFAT

IFAT was carried out for the detection of *T. annulata* antibodies in the serum samples as described by FAO [11]. Negative and positive *T. annulata* control sera were provided by the Central Veterinary Research Laboratory, Soba, Sudan. Examinations of the stained slides were carried out under 40× objective using Olympus Vanox incident-light excitation fluorescent microscope (Japan).

### Molecular detection

#### DNA extraction from whole blood samples

DNA was extracted from whole blood samples using DNA extraction kit (Qiagen, Germany) according to the manufacturer instructions. For quality assessment, 5 µl of extracted DNA was analyzed on 0.8% agarose gel.

#### Polymerase chain reaction (PCR)

*T. annulata* specific primers were used to amplify a 721 bp fragment from the gene encoding 30-KDa major *T. annulata* merozoite surface antigens (Tams 1) as described by d'Oliveira *et al*. [12]. The final reaction was performed in a volume of 25μl detailed as follows: 5 μl Green Master Mix, containing dNTPs, 1× PCR buffers (50 mM Kcl, 10 mM Tris-Hcl (pH=8), 0.5% Tween 20, 100 μg proteinase k per ml) and 3U Taq polymerase (intron), 13 μl H_2_O, 1 μl of each primer, and 5 μl genomic DNA. Thermocycler program was as follows: 94°C for 5 min, and then 30 cycles consist of 94°C for 1 min, 55°C for 1 min, and 72°C for 1 min, and final extension step at 72°C for 7 min, and hold on 4°C. In each run, positive control (DNA extracted from *T. annulata* cell line) and negative control (distilled water) were included. The amplified fragments were separated by electrophoresis on 1.5% agarose gel run at 80 V for 50 min, then visualized, and documented.

#### Tick identification

Ticks were identified according to Hoogstraal [13] and Walker *et al*. [14].

### Statistical analysis

Statistical analysis was performed using “Statistical Package for Social Science,” version 16. The mean and standard error of the results was estimated and then subjected to one-way analysis of variance to compare each factor with the results and to identify differences among sample means. Significance was considered when the probability is below 0.05. The 95% confidence interval (CI) was calculated.

## Results

### Blood smears

Of 150 blood smears, 11 (7.3%, 95% CI: 9.1-5.5) were positive for *Theileria* spp. piroplasm (Table-1). The highest prevalence rate was reported in Karary (12.5%), followed by Almawashi (10.5%) and Al shabi and Algeer (7.1%), and the lowest prevalence rate was in Al gabal (6.9%) followed by Museah (5.7%) (Table-1). No piroplasm was seen in Alseraif. There was no significant difference (>0.05) between Algeer, Alshabi, and Museah, while significant difference (p<0.05) between Karary and Alseraif was reported. The prevalence rate of *Theileria* spp. piroplasms in indigenous breed of cattle was higher (8.9%) than in crossbred (4.9%) (Table-2). Regarding age groups, the highest prevalence rate was 18.6% recorded among cattle 1–3 years old and the lowest prevalence rate was 0% recorded among cattle <1 year old (Table-2). The prevalence rates were 6.8% in females and 11.2% in males (Table-2).

**Table-1:** Distribution of *Theileria annulata* infection in Nyala according to three diagnostic techniques.

Location	Number examined	Diagnostic techniques		

		BS (%)	IFAT (%)	PCR (%)
Museah	53	3 (5.7) [6.5-4.9]*	20 (37.7) [42.5-31.7]	10/36 (27.8) [31.1-24.5]
Algabal	29	2 (6.9) [6.2-7.6]	16 (55.2) [55.2-49.4]	9/16 (56.3) [60.7-51.9]
Karary	16	2 (12.5) [13.5-11.5]	12 (75) [80.9-69.1]	7/14 (50) [53.7-46.3]
Almwashi	19	2 (10.5) [11.4-9.6]	11 (57.9) [62.8-53]	9/13 (69.2) [74.1-64.3]
Alshabi	14	1 (7.1) [6.9-7.6]	6 (42.9) [46-39.8]	2/10 (20) [21.2-18.8]
Algeer	14	1 (7.1) [6.9-7.6]	5 (35.7) [38.3-33.1]	2/11 (18.2) [19.4-17]
Alseraif	05	0	0	0
**Total**	150	11 (7.3) [9.1-5.5]	70 (46.7) [57.7-35.7]	100/39 (39) [46.6-31.4]

BS=Blood Smear, IFAT=Indirect Fluorescent Antibody Test, PCR=Polymerase Chain Reaction,

* [ ] = 95% Confidence Interval (CI)

**Table-2:** Comparison between the three diagnostic techniques used for detection of *Theileria annulata* infection among different breeds, age groups and both sexes of cattle in Nyala.

Parameters	Number examined	Diagnostic techniques		

		BS (%)	IFAT (%)	PCR (%)
Breeds				
Cross	61	3 (4.9) [5.7-4.2]*	20 (32.8) [37.8-27.8]	10/47 (21.3) [24.2-18.4]
Indigenous	89	8 (8.9) [10.6-7.2]	50 (56.2) [57.2-55.2]	29/53 (54.7) [62.5-46.9]
**Total**	150	11 (7.3) [9.1-5.5]	70 (46.7) [57.7-35.7]	39/100 (39) [46.6-31.4]
Age groups				
<1 year	20	0 (0.0)	3 (15) [16.3-13.7]	3/15 (20) [21.5-18.5]
13 years	58	6 (18.6) [20.1-17.1]	25 (43.1) [49.5-36.7]	17/37 (45.9) [51.4-40.4]
>3 years	72	5 (7.7) [8.9-6.5]	42 (58.3) [68-48.6]	19/48 (39.6) [45-34.2]
**Total**	150	11 (7.3) [9.1-5.5]	70 (46.7) [57.7-35.7]	39/100 (39) [46.6-31.4]
Sex				
Females	132	9 (6.8) [8.3-5.3]	62 (46.9) [57.9-35.9]	33/82 (40.2) [47.3-33.1]
Males	18	2 (11.1) [12-10.2]	8 (44.4) [48.1-40.7]	3/18 (16.7) [18.1-15.3]
**Total**	150	11 (7.3) [9.1-5.5]	70 (46.7) [57.7-35.7]	39/100 (39) [46.6-31.4]

BS=Blood Smear, IFAT=Indirect Fluorescent Antibody Test, PCR=Polymerase Chain Reaction,

* [ ] = 95% Confidence Interval (CI)

### IFAT

Of 150 serum samples, 70 (46.7%, 95% CI: 57.7-35.7) were positive for *T. annulata* antibodies by IFAT. Prevalence of *T. annulata* antibodies was highest in Karary (75%), followed by Almawashi (57.9%), Algabal (55.2%), and Al shabi (42.9%), while the lowest in Museah (37.7%) and Algeer (35.7%) (Table-1). There was no significant difference (>0.05) between Algeer, Museah, Alshabi, and Almawashi, and there was significant difference (p<0.05) between Karary and Algabal. The highest prevalence of antibodies against *T. annulata* was in indigenous breeds (56.2%) compared with the crossbreed (32.8%) (Table-2). The older group of cattle (>3 years old) recorded 58.3% prevalence rate as the highest prevalence of antibodies against *T. annulata* while the lowest rate (15%) was recorded among calves (<1 year old) (Table-2). The prevalence rates were 46.9% in females and 44.4% in males (Table-2).

### PCR

**Figure-2:**
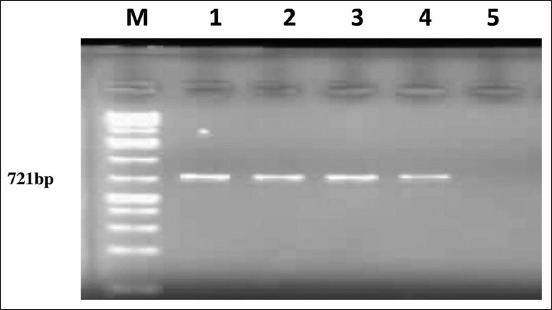
Agarose gel electrophoresis of *Theileria annulata* DNA amplified using a primer set N516/N517 (Tams-1). M: DNA-maker 100 bp DNA ladder. Lane 1: Positive control of *T. annulata*. Lane: 2-4 field samples. Lane: 5 water control.

A fragment of 721 bp was amplified in 39 (39% 95% CI: 46.6-31.4) of 100 samples (Figure-2). The prevalence of *T. annulata* was highest in Almawashi (69.2%), followed by Al gabal (56.3%) and Karary (50%), while the lowest prevalence rate was in Museah (27.8%), Al shabi (20%), and Algeer (18.2%) (Table-1). There was no significant difference between locations (>0.05). The prevalence was higher in the indigenous breed (54.7%) than crossbred (21.3%) (Table-2). The highest prevalence rate was 45.9% among cattle 1–3 years old, and the lowest prevalence rate was 20% recorded among calves (<1 year old) (Table-2). The prevalence values were 40.2% in females and 16.7% in males (Table-2).

## Tick survey results

The dairy cattle examined were found to be infested with three tick genera and ten different species of ticks, included *Rhipicephalus evertsi evertsi*, *Rhipicephalus decoloratus, Rhipicephalus annulatus*, *Hyalomma dromedrii*, *Hyalomma impeltatum*, *Hyalomma rufipes, Hyalomma anatolicum*, *Hyalomma truncatum*, *Amblyomma variegatum, and Amblyomma lepidum*. The highly prevalent species were *R. evertsi evertsi* (28.7%), *H. rufipes* (18%), *A. variegatum* (12.7%), *H. dromedrii* (10.7%), *H. impeltatum* (8%), *A. lepidum* (6%), *R. decoloratus* (5.4%), *H. truncatum* (4.7%), *H. anatolicum* (4%), and *R. annulatus* (2%).

## Discussion

TBDs of cattle remain an important impediment to livestock development in the Sudan [15]. The impact of ticks and TBDs in South Darfur State is tremendous due to the high abundance of ticks throughout different climatic zones of the region [16]. Recently, due to the progressive development of South Darfur State and rapid growing of human population in Nyala town, there is an increasing demand of milk and milk by-products. Many farmers introduced Friesian cattle and their crosses with indigenous breeds (Friesian × Kenana or Butana) from Central Sudan. However, TBDs are the main threat to these cattle in most locations within the town.

A total of 150 blood samples were randomly collected from dairy cattle in Nyala town. The sensitivity of blood smear examination in the detection of *Theileria* parasites in blood smears was low, and only 11 (7.3%) of 150 blood smears were positive for *Theileria* spp. piroplasms. Salih [17] reported 3.9%, Abdallah [18] reported 2.8%, and Jaafer [10] reported 3.6% in the same region. Ali [19] reported 16.6% as the prevalence of *Theileria* spp. piroplasm in Khartoum State. Species differentiation using blood smears is not possible [1].

The seropositivity for *T. annulata* antibodies was 70 (46.7%) of 150 serum samples. It is higher than that recorded by Salih [17] who found 9.8% positive *T. annulata* antibodies and Jaafer [10] who reported 31% using IFAT. The gradual increase in the incidence may be due to the introduction of dairy cattle from Central Sudan without regulations imposed to eradicate ticks from cattle where it acts as a vector for the spread and increased prevalence of the disease in the region. It is also may be due to the introduction of the vector *H. anatolicum* with the exotic breeds of cattle that the disease has become widespread.

Blood smears examination revealed lower sensitivity in detection of *T. annulata* compared with serological tests. Thus, piroplasms were detected in 7.3% while 46.7% *T. annulata* antibodies were positive by IFAT. This finding is in agreement with the previous studies [20] which found that blood smears revealed 13.8% piroplasms and seropositivity of 33.3% working on *T. annulata* using BS, IFAT, and ELISA to determine the epidemiology of tropical theileriosis. Ali [19] reported that blood smears revealed the lowest sensitivity in detection of carrier cattle to *T. annulata* compared to PCR. PCR improved the sensitivity of detection of the parasite and 39/100 (39%) had detectable parasite DNA, while IFAT shows 46.7% prevalence. Keeping in mind that the PCR is detecting the parasite DNA, while IFAT targeting the antibodies against the parasite. The results agreed with the previous studies which indicated the higher sensitivity of PCR-based techniques compared with the other diagnostic techniques such as microscopic examination in the diagnosis of *T. annulata* [12]. Ali *et al*. [21] reported that a total of 162 field samples obtained from cattle in Khartoum State tested for *T. annulata*, 27 (16.6%) were positive in blood smears, and 78 (48%) were positive by PCR technique using primer specific for the gene encoding of the 30 kDa major merozoite surface antigen of *T. annulata* [12].

Concerning location, the result showed no significant difference (>0.05) between seven locations, and all these locations were not far from each other inside Nyala.

The prevalence rate of *T. annulata* infection in zebu cattle was higher than the crossbred in three diagnostic techniques. This is probably due to the application of acaricides to control ticks on exotic breeds besides the fact that exotic breeds are reared under good management systems, high level of nutrition, maintenance of good hygiene conditions, and fairy proper veterinary supervision. Ali *et al*. [21] reported low level of piroplasms by blood smears but high prevalence of *T. annulat*a parasites using PCR and RLB indicating that most of the animals were carriers of *T. annulata*. This could hold true in this study since the prevalence of *Theileria* spp. piroplasms was lower in Zebu cattle. In Sudan, indigenous cattle are normally resistant to TBDs, but they may be severely affected or even dies if stressed [22]. Bakheit and Latif [23] demonstrated significant differences in disease resistant to the indigenous breed Kenana compared to Friesian cattle. Salih *et al*. [6] compared zebu and crossbred (Zebu×Friesian) and detected differences in a relative risk. In the current study, 56.1% of apparently healthy Zebu cattle had antibodies against *T. annulat*a, while 54.7% positive by PCR, and only 8.9% of them showed *Theileri*a spp. piroplasms in the blood smears. The results also agreed with El Hussein *et al*. [24] and Salih [17]. They found that *T. annulata* infection was more prevalent in indigenous animals. The molecular techniques are more effective in detecting *T. annulata* infection and have higher specificity in comparison with other techniques [25].

Three genera and ten species of ticks were found infesting cattle in Nyala. This result is similar to previously reported in Darfur by Osman [22]. This author had reported twenty tick species in the same region on domestic animals under nomadic systems. Recently, Abdallah [16] recorded 15 tick species from sedentary and semi-sedentary cattle in South Darfur State, and Jaafer [10] recorded 13 tick species from semi-intensive and extensive systems in and around Nyala dairy cattle. However, there was a difference in tick abundance according to different animal husbandry type. The current study revealed lower tick load on dairy cattle in Nyala. This may be attributed to the control of ticks by chemical acaricides in these farms, especially in crossbred farms.

## Conclusion

The study concluded that tropical theileriosis is prevalent among dairy cattle in Nyala. *H. anatolicum* was found in very low numbers, suggesting that other ticks may play a role in the transmission of the disease. Molecular characterization of *T. annulata* is recommended for accurate mapping of the disease and evaluates the magnitude problem of tropical theileriosis in South Darfur region.

## Authors’ Contributions

The present study was a part of IAA’s original research work which includes study design, collection of blood, serum and tick’s samples, examination of blood smear, identification of tick species and statistical analysis, preparing, and drafting the manuscript during M.V.Sc. thesis program. DAS had designed the plan of work as well as provided guidance during the entire experiment and corrected manuscript. LME, REA, and MMO helped during IFAT procedure. AMA was the conceived of the study providing supervision and statistical analysis and manuscript preparation. All authors read and approved the final manuscript.
